# Development and validation of a sperm-freezing device created using 3D printer technology

**DOI:** 10.5935/1518-0557.20240105

**Published:** 2025

**Authors:** Vera Lucia Lângaro Amaral, Gabriela Reif, Rafael Alonso Salvador, Cleiton Alves de Oliveira, Alfred Paul Senn, Tiago Góss dos Santos

**Affiliations:** 1 Universidade do Vale do Itajaí, Itajaí, Santa Catarina, Brazil; 2 Department of Genetic Medicine and Development, University of Geneva, Geneva, Switzerland; 3 A.C.Camargo Cancer Center, São Paulo, Brazil

**Keywords:** sperm, cryopreservation, liquid nitrogen vapor, 3D printer, reproducibility

## Abstract

**Objective:**

To develop and evaluate the effectiveness of a 3D-printed prototype to hold semen straws during the freezing process under safe and reproducible conditions.

**Methods:**

A prototype capable of holding ten straws in liquid nitrogen vapor (LN2) was 3D printed. A second support that is commonly used was assembled from pieces of expanded polyethylene (EPS), respecting the identical distance between the straws and the LN2 surface. Temperatures were registered with a thermocouple placed inside a straw. Semen samples were frozen in the presence of cryoprotectant using the prototype (n=20) and the EPS support (n=20) in two independent series of measurements. Sperm parameters (motility, vitality, and DNA fragmentation) were measured for fresh and frozen-thawed samples.

**Results:**

The temperature cooling curves measured on the prototype were remarkably reproducible. The prototype material withstood over 300 freezing cycles without damage. The mean motility and vitality of fresh (64.2%, 72.0%) and frozen-thawed (25.7%, 38.8%) samples were significantly different (*p*<0.001) using either support. Recovery rates of motility, vitality, and sperm DNA fragmentation in frozen-thawed sperm samples were equal regardless of straw position on the prototype or support type used.

**Conclusions:**

The developed device allows a homogeneous, quantifiable, reproducible cooling of the straws in liquid nitrogen vapor. The recovery rates are comparable to those reported in the literature for both tested supports. The designed 3-D printed prototype favors the safe handling of the straws, an explicit way of describing freezing conditions, and a better intra-operator and inter-laboratory reproducibility of the cryopreservation process.

## INTRODUCTION

Sperm cryopreservation is a widely used technique in assisted reproduction (ART) that has resulted in the birth of millions of babies worldwide (Tao *et al*., 2020). Cryopreservation protocols described in the literature differ regarding cooling rate, cryoprotectant composition, sample packaging, and thawing method (Li *et al*., 2019). Although these protocols give satisfactory results, research on which one gives the best live sperm recuperation rate remains ongoing (Huang *et al*., 2022; Zhou *et al*., 2021). For some authors, it is not only the optimal rate that should be considered but also the inter-individual and inter-species differences (Holt, 2000; Nikitkina *et al*., 2022).

Currently, different methods are used to cryopreserve human semen, programmable (slow) freezing, manual methods, which may feature different cooling ramps, and the vitrification technique applicable only for small volumes (Hinting & Agustinus, 2020). Slow freezing, based on stepwise cooling, is a widely used routine procedure (Tao *et al*., 2020; Tongdee *et al*., 2015). The programmable method allows more precise control of these cooling ramp rates using computer-controlled electric valves. However, this expensive method is only cost-effective for processing many samples.

Rapid freezing is based on direct contact of samples with liquid nitrogen LN2 vapor for 10-15 minutes (Li *et al*., 2019). It can support different types of sample packaging, requiring only one cryopreservation step, which reduces the operation time and increases cost-effectiveness. On the other hand, temperature control remains manual, and the distance of samples from the LN2 surface is not standardized (Di Santo *et al*., 2012; Rios & Botella, 2019). Rapid freezing offers higher post-thaw motility and survival rate than slow freezing (Riva *et al*., 2018).

Finally, vitrification is a fast, simple, and low-cost cryopreservation method, but it requires micro volumes (≤20 µl), which makes it less attractive when dealing with large-volume samples (Tao *et al*., 2020). This technique allowed the birth of the first child in 1953 following freezing on dry ice of a sperm-glycerol mixture in droplets (Bunge & Sherman, 1953).

Regardless of technique, cryopreservation impairs sperm viability through mechanical and osmotic damage at the cellular or subcellular levels (Di Santo *et al*., 2012; Hinting & Agustinus, 2020; Zribi *et al*., 2010). The effect of freezing on sperm DNA fragmentation is still disputed, as it is sensitive to confounding parameters, such as the freezing protocol used, the quality of the seminal sample, the method of sperm preparation, or the DNA fragmentation assessment test (Isachenko *et al*., 2004a).

In the case of manual cryopreservation, the critical step is the exposure of the samples to liquid nitrogen vapor. Often, the distance of the samples from the liquid nitrogen surface or the exposure time is not reported in the literature, or when they are described, they are not very explicit. Thus, there is a gap in the description of this crucial step, giving freedom to any user to create his means and rules. As a result, this methodology differs from one laboratory to another, and the rate of viability recuperation of samples can vary greatly.

This study aims to determine whether developing a three-dimensional printed prototype using thermoplastic can increase the cryopreservation step standardization, efficiency, and reproducibility while allowing modifications to the device that would meet different cryopreservation protocols.

## MATERIALS AND METHODS

### Ethical approval

This study was submitted to the Research Ethics Committee of the Universidade do Vale do Itajaí (UNIVALI, SC, Brazil), which accepted it under C.A.A.E. No. 39941420.2.0000.0120. The volunteers had a mean age of 35.9 (min-max: 25-46), and all signed an informed consent form before enrollment.

### Preliminary inquiry among laboratories performing sperm cryopreservation

An online questionnaire (Google Forms) was prepared to learn the methodologies routinely used in assisted human reproduction clinics and sperm banks. The link to this form was sent in January 2021 via WhatsApp to a group of approximately 200 embryologists. The results showed that of the 36 responses obtained, 83% indicated using a freezing system in the LN2 vapor stage. Regarding the holder used for the process, 58.3% responded that it was improvised manually, 44.4% are made of metal, 36% of expanded polystyrene (EPS), 14% of polypropylene, or other materials (6%).

### Development of the cryopreservation prototype

**Figure 1:**
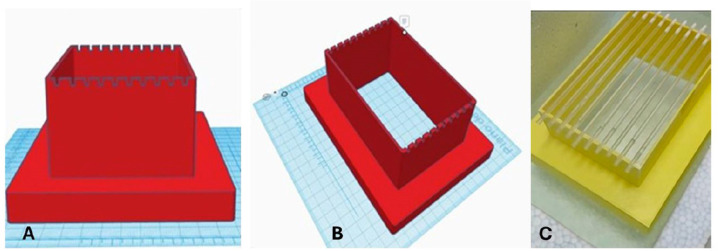
Views of the straw holder, consisting of a hollow parallelepiped resting on a floating base. A/B: Three-dimensional projection of the prototype, and C: 3D-printed straw holder. The indentations allow ten straws to be placed securely in the horizontal position.

A prototype consisting of an empty parallelepiped measuring 12x8x5 cm (LxWxH), superimposed on a floating base 16x12x1.5 cm (LxWxH), was designed in a three-dimensional modeling program (AUTODESK, Tinkecard) and 3D printed with acrylonitrile-butadiene-styrene (ABS) filaments as seen in Figure 1. This prototype can accommodate ten straws fixed horizontally by indentations numbered 1 to 10. When floating on LN2, the immersed part of the prototype does not exceed 1.5 cm, so the straws remain at a height of 5 cm. Successive immersions in LN2 verified the durability and strength of the support, and the ABS material showed no changes in texture, shape, or stiffness after more than 300 freezing cycles. The safety of handling the straws during the freezing process and the possibility of sanitization after each use were also confirmed.

### Hand-made EPS support

**Figure 2:**
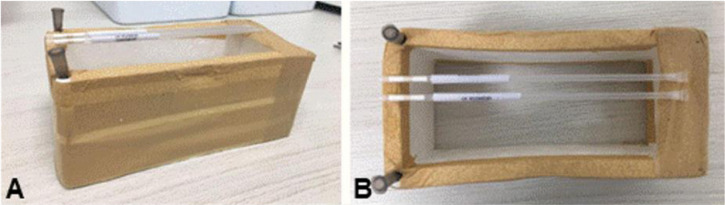
Hand-made device made of expanded polystyrene (EPS) used to support straws with seminal samples for floating in N2L vapor during cryopreservation. Side view on A and top view on B.

An EPS support was made in the shape of a hollow parallelepiped, with dimensions 12 x 8 x 5.5 cm (L x W x H), covered with insulating tape to increase its durability (Figure 2). The EPS support floats well over LN2, and the submerged portion does not exceed 0.5 cm, so the straws stay at 5 cm over the LN2 surface.

### Semen collection and analysis of initial parameters

Seminal samples (n=40) were collected between 2021 and 2022 in an andrology laboratory in Itajaí/SC. Twenty samples were used to test the position of the straws in the prototype (Test #1), and 20 samples were used to compare the ABS with the EPS support (Test #2). Ejaculates were obtained by masturbation after 2-5 days of abstinence and immediately incubated at 37°C until complete liquefaction. Sperm concentrations were determined using a Makler counting chamber, and motilities were determined under a microscope at 400x magnification and classified as progressive, non-progressive, and immotile. Vitality was determined after mixing equal parts of sperm and eosin isotonic dye (0.5%) by microscopic observation (400x) of the preparations under a coverslip. The vitality was expressed as the percentage of sperm without staining. For morphology, fresh semen smears were stained by DiffQuick (Panotic, Laborclin, Brazil), and 200 spermatozoa were observed under a microscope (1000x). World Health Organization recommendations were followed for semen analysis (WHO, 2021).

### Sperm DNA fragmentation analysis

The fragmentation rate of sperm DNA was determined by the chromatin dispersion method (SCD) adapted from (Fernández *et al*., 2003). Semen samples were diluted in a culture medium (GV-HEPES, Ingámed, Maringá, Brazil) to reach a final sperm concentration of approximately 15x106/mL. Microscopic slides were first pre-coated with a 0.65% agarose solution (Sigma Aldrich, São Paulo, Brazil). Subsequently, 70 µL of semen was mixed with 30 µL low melting point agarose (Sigma Aldrich, São Paulo), and 30 µL of this mixture was placed on the agarose pre-treated slide and covered with a coverslip. The mounted slide was cooled for five minutes at 4°C before the coverslip was gently slid away.

The slide was held horizontally, and the sperm-agarose mixture area was covered with a few drops of a denaturation solution (0.08N HCl) for 7 min. The excess liquid was then allowed to be drained away by holding the slide vertically. The exact sequence of manipulation was then followed using lysis solution A (0.4 M Tris-HCl (Sigma-Aldrich, São Paulo, Brazil), 0.8 M DTT (Sigma-Aldrich, São Paulo, Brazil), 1% SDS (AppliChem, Germany), 50mM EDTA (Uniscience, São Paulo, Brazil), pH 7.5) for 10 min, lysis solution B (Tris-HCl 0.4 M, SDS 1%, NaCl 2M, pH 7.5) for 5 min, wash solution (PBS, São Paulo, Brazil) 5 min, and an alcohol series (70%-100%).

The slides were allowed to dry in ambient air before being stained with DiffQuick (Panotic, Laborclin, Brazil), and 200 spermatozoa were observed and graded under a microscope (1000x).

The extent of the formed halo was used to detect fragmentation. Spermatozoa with fragmented DNA were those with no halo, degraded head appearance (fragmented or with very weak staining), or small halos (≤ 1/3 of the head diameter). Non-fragmented sperm were those with a large (≥ 3x of the head diameter) or medium halo (> 1/3 and < 3x of the head diameter).

The sperm DNA Fragmentation Index (DFI) was calculated as follows:

DFI (%) = (No. fragmented spermatozoa / No. counted spermatozoa) * 100

### Temperature monitoring

**Figure 3:**
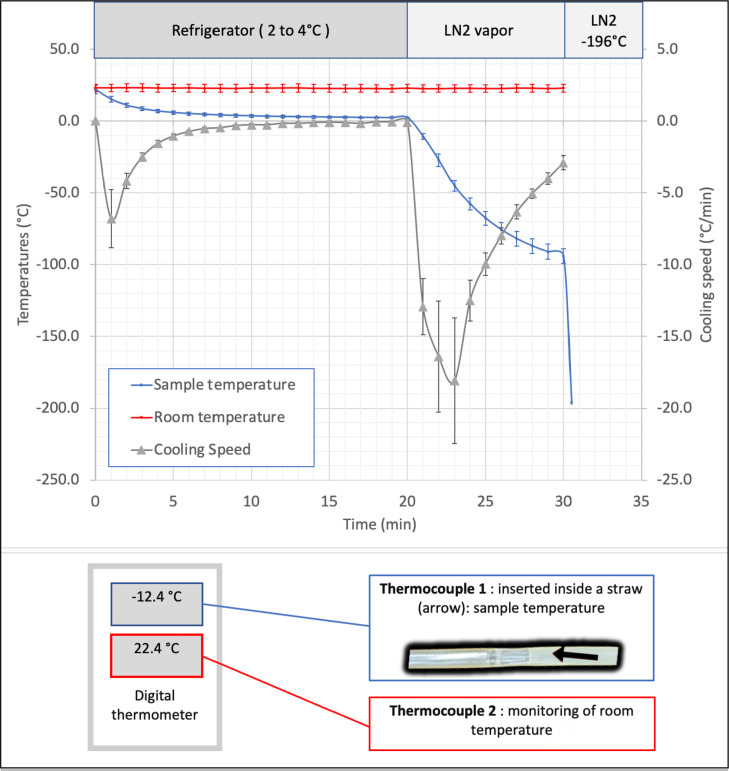
Temperatures recordings by two thermocouples (Akzo Instrumentos, Brazil), one inserted in a control straw in position 5 of the device and the second in ambient air. Three cooling periods are monitored: 1) for 20 min in a refrigerator, 2) for ten minutes in an expanded polystyrene container containing liquid nitrogen, the support is then in fluctuation mode, 3) for a variable period in liquid nitrogen (LN2) the straws being then released from the support.

The temperatures of the samples and the ambient air of the laboratory were measured throughout the freezing process using two thermocouples (Akso^®^, Akso Instrumentos de medição, Brazil), certified and calibrated, one placed in the ambient air of the laboratory, the other in one of the straws containing a sample of sperm diluted with the cryoprotectant and placed in position 5 on the device. Six series of measurements were made over several months. The measured temperatures were highly reproducible, regardless of the position of the straw containing the thermocouple on the holder (Figure 3) or external variations of the laboratory temperature.

### Test #1: Cryopreservation of samples located in different positions on the prototype

The samples were first analyzed at an andrology laboratory (Criovita, Itajaí) for routine semen analysis. They were then transported to the Laboratory of Reproductive Biotechnology (Univali), where the prototype’s freezing tests were performed. The transport time was approximately 15 min while samples were kept at ambient temperature (20-30°C) in closed conical tubes in the dark. Upon arrival, semen samples were diluted (1:1) with a culture medium (GV-HEPES, Ingámed, Maringá, Brazil) in conical tubes and centrifuged (300 g, 10 min). After removing the supernatant, the sediments were resuspended in 600 µL of the same GV-HEPES medium, and the parameters motility, vitality, and DNA fragmentation were analyzed.

The washed semen samples were diluted with freezing medium (Ingá Sperm Freezing, Ingámed, Maringá, Brazil), containing egg yolk and glycerol, in the proportion 1:1, and packaged in 3 straws of 0.5 mL. The previously identified straws were placed in positions 1, 5, and 10 in the prototype. Three cooling periods were imposed by transferring the prototype for 20 minutes to a refrigerator (4±2°C), then for 10 minutes to a styrofoam box measuring 15.5 x 13.7 x 27.5 cm (L x W x H) containing approximately 750 mL of LN2, with the prototype in floating mode. At the end of these operations, the straws were released from the support and immersed in LN2 before being transferred to a cryogenic cylinder. At the end of the cryopreservation period (≥24 h), the straws were removed from the cryogenic container and kept for 20 min at 28°C. The thawed samples were washed, and the sediments were resuspended in 400 µL of GV-HEPES. Motility and vitality parameters were analyzed, and sperm DNA fragmentation was checked.

Recuperation rates R (%) were calculated for each sample as the post-thaw over pre-thaw ratios of the measured values.

R (%) = (Measure) Post Thawing / (Measure) Pre-Freezing X 100.

The value Measure is either total motility (%) or vitality (%).

### Test #2. Cryopreservation of samples using the ABS prototype vs. EPS straw holder (Control)

The samples (n=20) were cryopreserved using Test-Yolk Buffer diluent medium (TYB^®^, Irvine Scientific, USA) in a 1:1 ratio, and the content was filled into four correctly identified 0.5 mL straws. Two straws were inserted in the ABS prototype in regions 5 and 6, near the center, while the other two were placed in the EPS support. The cooling steps were performed in an equal manner for both supports by placing both supports for 20 minutes in a refrigerator (4±2°C), then in an EPS box of 15.5 x 13.7 x 27.5 cm (L x W x H) containing approximately 750 mL of LN2, where the supports remained floating while keeping the straws exposed to LN2 vapor for 10 minutes. The straws were then released, immersed in LN2, and transferred to a cryogenic tank for at least 24 hours.

The straws were removed from the cryogenic tank and left to thaw at 37°C for 20 minutes. The samples were diluted 1:1 in the GV-HEPES medium. The samples were centrifuged at 300 g for 5 minutes, the supernatants were removed, and the sediments were resuspended in 500 µL of GV-HEPES. The samples were kept at 37°C for 10 minutes, then motility, vitality, and sperm DNA fragmentation analyses were performed. After the analyses, the samples were kept at 37°C for a survival test over 24h. The same sperm parameters were then analyzed again.

### Statistical analysis

The data were stored in an Excel spreadsheet. Jamovi statistical software (version 1.6.23.0, Sydney, Australia) was used for the statistical evaluation of the data. ANOVA and STUDENT t-tests were used to analyze continuous variables (motility, viability, fragmentation). Differences were considered statistically significant for *p*-values <0.05.

## RESULTS

### Characteristics of seminal samples used in Test #1: straw positioning

The seminal characteristics, as mean values ± standard deviations, of the sperm samples used in Test #1 and Test #2 are shown in Table 1, together with the corresponding reference values (WHO, 2021).

**Table 1:** Values (mean ± standard deviation) of the main initial seminal parameters of the samples (n=40) used for Test #1 and Test #2.

Parameters	Test #1 Mean ± SD	Test #2 Mean ± SD	WHO ref.^*^
Volume (mL)	3.6±1.6	3.3±1.9	≥ 1.4
pH	7.8±0.4	7.6±0.5	≥7.2
Concentration (x10^6^/mL)	58.8±37.2	61.1±27.6	≥16
Total sperm count (x10^6^)	188.9±113.7	201.6±120.4	≥42
Progressive motility (%)	51.5±8.6	40.4±5.2	≥30
Non-progressive (%)	22.1±4.8	24.7±5.2	≥1
Total motility (%)	73.6±6.3	65.2±6.3	≥42
Vitality (%)	78.6±5.4	71.2±1.4	≥54
Normal morphology (%)	4.5±1.4	4.3±1.3	≥4

* WHO (2021): 5th centile.

### Temperature measurements during the cooling process with the prototype

The ambient temperature of the laboratory and the temperature inside a straw placed in position 5 of the freezing device were measured during six experiments using two thermocouples. The values recorded are presented in Figure 3. Three steps are to be distinguished: 1) the passage in the refrigerator at a temperature between 2-4°C; 2) the transfer of the device in fluctuation on LN2 in an expanded polypropylene box with a lid, the straws being then at a distance of 5 cm above the surface of the nitrogen; 3) the deposition of the straws in the liquid nitrogen The temperature variations in these three situations show an asymptotic decrease in temperature towards an equilibrium point: +2°C for phase 1 after 30 min, -99°C for phase 2 after 20 min, -196°C for phase 3 after <1 min. Based on the observation that these curves were remarkably reproducible, temperature monitoring was not systematically performed when freezing the samples in the following experiments.

### Determination of sperm motility and vitality before and after freezing (Test#1: straw positioning)

Motility and vitality parameters were measured before and after the seminal samples’ thawing. The values are reported in Table 2, and significant differences were observed for both parameters. The data were also analyzed considering the position of the straws in the prototype during freezing. Motility and vitality before freezing and after thawing for the three positions 1, 5, and 10 are reported in Table 3A. No statistical difference was observed between the three positions. Recuperation rates varied between 28% and 45% for motility and 52% and 55% for vitality. The observed variations are not significant (*p*>0.3). Motility and vitality recuperation rates using results from all straws frozen with the prototype are shown in Table 3B. Sperm DNA fragmentation rates were not modified by the freeze-thaw procedure (Table 2).

**Table 2:** Values (mean ± standard deviation) of progressive, non-progressive motility, immobile forms, vitality, and sperm DNA fragmentation index (DFI) pre-freezing and post-thawing.

Semen parameters	Before freezing		After thawing		p
	Mean ± SD	Median (Min, Max)	Mean ± SD	Median (Min, Max)	
Progressive motile (%)	40.4±10.1	39.8 (12.5, 55)	12.7±9.6	10.7 (2.7, 35)	<0.001
Non-progressive (%)	23.8±4.2	23.5 (16.5, 34)	13.0±4.4	12 (7, 23.5)	<0.001
Total motility (%)	64.2±8.8	64.3 (40.5, 80.5)	25.7±13.1	21.7 (9.7, 49.7)	<0.001
Immotile (%)	35.8±8.8	35.8 (19.5, 59.5)	74.4±13.1	78.3 (50.3, 90.3)	<0.001
Vitality (%)	72.9±8.2	73.3 (54, 85)	39.5±9.7	38.8 (22.3, 53.7)	<0.001
DFI (%)	26.1±9.6	27.8 (10.6, 40.4)	27.1±14.2	22 (10.6, 53)	NS

**Table 3A:** Total motility, vitality pre-freezing and post-thawing, and recuperation rates (R) according to the position of the straws (1,5 and 10) in the prototype. Values are means ± standard deviations.

Parameters	Position in device			p
	1	5	10	
Motility pre-freezing	63.1±13.4	64.3±10.6	64.7±5.9	>0.3
Motility post-thawing	18±7.3	25.4±13.6	29.6±14.4	>0.3
R total motility	28.3±7.4	38.5±19.3	45.4±21.4	0.35
Vitality pre-freezing	71.6±10.3	72.4±7.9	73.8±8	>0.3
Vitality post-thawing	37.4±9	39.2±10.3	40.6±10.7	>0.3
R vitality	52.1±8	53.9±11	54.9±13	0.76

**Table 3B:** Recuperation rates (R) of motility and vitality using results from all straws frozen with the prototype. Values are means ± standard deviations.

Parameters	Recuperation rates (%)	
	Mean ± SD	Median (Min, Max)
Motility	39.4±18.9	33.2 (18.8, 78.2)
Vitality	53.9±10.9	53.5 (34.2, 73.5)

### Determination of sperm motility and vitality pre-freezing and post-freezing (Test#2: ABS *vs*. EPS)

**Figure 4:**
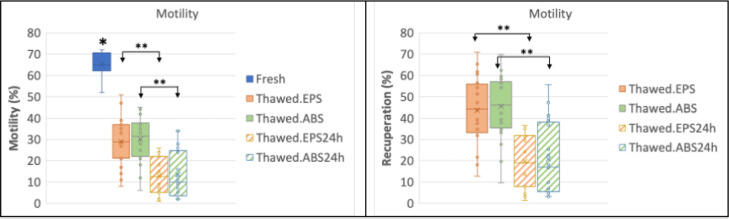
Boxplot of the motility of fresh, thawed, and after 24h of culture at 37°C, using EPS support and ABS prototype, and recuperation rates (R)

As visualized in Figure 4, an average initial total motility of 65.5% was found in the fresh sample and 71.2% in the vitality. It showed a significant difference between motility before cryopreservation and after thawing in both groups (*p*<0.001). There was no significant difference when the two thawed groups were compared (*p*=0.7).

* Fresh semen is significantly (*p*<0.001) different from the other groups.

** Significant difference (*p*<0.001) after thawing and 24hs of culture.

Analysis by paired samples test-test.

**Figure 5:**
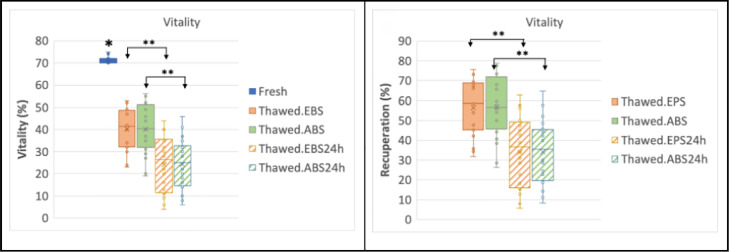
Boxplot of the vitality of fresh, thawed, and after 24h of culture at 37°C, using EPS support and ABS prototype, and recuperation rates (R)

After thawing, sperm motility recuperation rates of 43.6% were obtained for the group cryopreserved using the EPS support and 45.5% for the group of frozen samples using the ABS prototype. The vitality recuperation rate was 56.4% for the EPS and 56.6% for the ABS prototype; no significant difference was found between the two (*p*=0.9). The recuperation rates of vitality and sperm motility of ABS and EPS groups also showed no significant difference after cultivation for 24 hours at 37°C (Figures 4 and 5).

* Fresh semen is significantly (*p*<0.001) different from the other groups.

** Significant difference (*p*<0.001) after thawing and 24hs of culture.

Analysis by paired samples test-test.

### Sperm DNA fragmentation before and after cryopreservation in ABS Prototype *vs*. EPS support

The average DFI of the fresh samples was 11.56%. After thawing, a significant increase in DFI was observed when using both the EPS (25.1%) holder and the ABS prototype (24.9%). Both frozen groups showed significant differences (*p*<0.001) when compared to the fresh sample but showed no difference between each other (*p*=0.8). The DFI verified after 24 hours of culture at 37°C showed no difference between the EPS and ABS groups (Table 4).

**Table 4:** Sperm DNA fragmentation index (DFI) after thawing and 24 h of culture. Equal letters are used to identify statistically significant differences with paired-sample t-tests.

	DFI (%)	DFI 24h (%)
Fresh semen	11.6±9.4^*^,^†^	ND
Thawed EPS	25.1±16^*^	30.7±16.4
Thawed ABS	24.9±18.1^†^	27.6±15.9

* ,^†^: *p*<0.001.

## DISCUSSION

In the framework of our study, we have developed a prototype using a 3D printer capable of ensuring the freezing of sperm straws. The ABS polymer combines properties beneficial for our projects, such as stability in a wide range of temperatures, low thermal conductivity, high resistance to impact and chemical aggression, resistance to deformation, and low mass density (Vishwakarma *et al*., 2017). The prototype was found to be able to float on liquid nitrogen and withstand more than 300 freeze-thaw and sanitization cycles without structural alteration, the appearance of cracks, or deformations. This prototype also has a low thermal conductivity, contrary to metal supports which induce heterogeneous thermal exchanges and do not allow for control of the distance of the straws above the LN2 surface. It also solves the problem of lower durability of polystyrene-based flotation systems, which have a high negative ecological impact, are difficult to sterilize, and do not allow standardization of their three-dimensional structures. Furthermore, thanks to 3D printing technology, various types of devices can be produced to suit specific needs by varying the height, number, or shapes of the sample holders while maintaining a positive cost-benefit ratio.

An essential objective of our study was to measure the temperature drop when the device was placed in a refrigerator at 2-4°C and then in the vapor phase of liquid nitrogen. The dimensions of the box where the LN2 vapor exposure occurred and the amount of LN2 initially present were normalized. The immersed portion of the floating device did not exceed 1.5 cm, which placed the straws at a height of 5.0±0.5 cm. The temperature recorded at this height was -99°C after 10 min of equilibration. Once the critical zone of -15°C to -60°C is passed, sperm metabolism ceases, and the risk of cryogenic damage is low enough that they can be immersed in LN2 (Graham, 1996). As shown in Figure 3, the standard deviations of the temperatures measured in the vapor phase of LN2 are more significant than those observed in the refrigerator, a fact that is associated with exothermic processes caused by crystallization. However, in our device, the coefficients of variation of the cooling rates remained <10%. Using the device allows thus the standardization of the freezing process, reducing its uncertain consequences on the spermatozoa.

When comparing motility recuperation for straw frozen at various positions in the prototype, we chose to work with samples washed by centrifugation with GV-HEPES nutrient medium. The survival rates obtained after thawing (Tables 3A and 3B) showed equal values regardless of the position of the straw in the prototype. The average motility recuperation rate after thawing was 39.4, within the 30-46% range reported by other authors (Oberoi *et al*., 2014; Saleh *et al*., 2018). Sperm motility can reduce by 25% to 75% after cryopreservation (Martins *et al*., 2019).

In our study, sperm motility and vitality evaluations were performed after a 20 minutes incubation at 37°C after thawing and removal of the cryoprotectant, as an increase in sperm motility has been shown to occur during this period (Oberoi *et al*., 2014). This increase was attributed to transient mitochondrial damage and the time needed to regenerate ATP. For vitality, the recovery of 53.9±10.9% was within the range of 45% to 52.5% found in the literature (Bandularatne & Bongso, 2002; Ozkavukcu *et al*., 2008; Watson, 2000).

In Test#2, the results obtained with the prototype and the EPS support, respectively, were similar for sperm motility (45.5% and 43.6%) and vitality (56.6% and 56.4%)recuperation. The observed decrease in sperm motility can be explained by the chemical and physical stress experienced by the spermatozoa during cryopreservation, such as intraand extracellular ice crystal formation, cell dehydration, and osmotic shock (Di Santo *et al*., 2012; Oberoi *et al*., 2014). The plasma membrane alterations, lipid peroxidation, oxidative stress, and sperm DNA damage affect cell longevity and performance (Fernandez & O’Flaherty, 2018; Lee *et al.*, 2017; Mazzilli *et al*., 1995). In our study, the survival test at 24h showed no significant difference between the two straw supports, as shown in Figure 4 and Figure 5.

The recovery of motion characteristics can be improved by selecting motile cells through swim-up before cryopreservation. The final viability and motility of the spermatozoa depends not only on the cryoprotectant used or the freezing method but also on the quality of the seminal sample (Esteves *et al*., 2000).

Temperature variations during the cooling and heating phases cause an increase in reactive oxygen species (ROS) (Gualtieri *et al*., 2021). These ROS can affect the polyunsaturated fatty acids in the membrane, causing lipid peroxidation and extravasation of intracellular enzymes, consequently reducing sperm vitality and motility and inducing sperm DNA fragmentation (Bucak *et al*., 2008; Zribi *et al*., 2010). Although DNA fragmentation may not prevent oocyte fertilization, a high level of fragmentation (>30%) leads to embryo apoptosis and miscarriage (Alvarez Sedó *et al*., 2017; Robinson *et al*., 2012).

In our study, the DFI was comparable in samples before freezing and after thawing in Test #1 (Table 2). It was also similar between the two types of supports (Table 4). Such stability of the fragmentation index was also observed in studies using conventional freezing (Duru *et al*., 2001; Isachenko *et al*., 2004b; Paasch *et al*., 2004), in vapor or slow freezing (Wongkularb *et al*., 2011), with various cryoprotectants (Raad *et al*., 2018), or in young cancer patients (Li *et al*., 2020). The situation is more controversial when washed sperm is being used, with some authors finding an increase in DFI after thawing (Thomson *et al*., 2009) while others do not (Jackson *et al*., 2010). This effect can be understood considering seminal processing techniques eliminate bacteria, deficient, poorly motile, and dead sperm, potential ROS producers (Malvezzi *et al*., 2014; Ruiter-Ligeti *et al*., 2017). Immature spermatozoa in the ejaculate may increase ROS production and induce differential DNA fragmentation between samples or selection techniques (Gil-Guzman *et al*., 2001). The survival test of 24h did not significantly alter these DFI (Table 4). Again, some studies do not show alteration, and others do (Ortiz *et al*., 2017).

The controversies between the studies can be explained by several reasons, such as the difference in the freezing techniques used, the number of samples used, the distinct semen preparation techniques before cryopreservation, and the different techniques for sperm DNA evaluation (Di Santo *et al*., 2012; Isachenko *et al*., 2004a). From a clinical point of view, the temporal stability of the DFI measured is a diagnosis aid in infertile men and should help select the decision-making process (Esteves *et al*., 2021, 2022).

In conclusion, the path to standardization of the cryopreservation technique requires an accurate, standardizable, and safe methodology. The developed prototype allows homogeneous and quantifiable freezing conditions of the samples in NL2 vapor, favoring higher intraand inter-laboratory reproducibility. Results obtained using the prototype are comparable to those of a homemade EPS support, which has the significant disadvantage of being more fragile, difficult to replicate, less sustainable, and environmentally unfriendly. The prototype may cryopreserve spermatozoa from ejaculates, epididymal or testicular micro-aspirates, or more extensive testicular biopsies. The flexibility of 3D printing allows specific needs related to the number of straws or container sizes to be easily covered.
